# Primary Cervicomedullary Hemangioblastoma with Extensive Caudal Cystic Extension into the Cervical Cord: A Case Report and Literature Review

**DOI:** 10.1055/a-2920-0717

**Published:** 2026-08-03

**Authors:** Shubham Singh, Ravi Shankar Prasad

**Affiliations:** 1 Department of Neurosurgery 30117 Institute of Medical Sciences, Banaras Hindu UniversityVaranasiUttar PradeshIndia

**Keywords:** hemangioblastoma, cervicomedullary junction, brainstem neoplasm, cystic extension, microsurgery, case report, literature review, VHL

## Abstract

**Background** Hemangioblastomas are benign, highly vascular World Health Organization (WHO) grade 1 tumors that most commonly arise in the cerebellum. Brainstem involvement is uncommon, and lesions originating from the cervicomedullary junction with extensive caudal cystic extension into the cervical spinal cord are exceptionally rare, posing significant diagnostic and microsurgical challenges.

**Case Description** A 39-year-old man presented with a 2-year history of progressive paresthesia involving both upper limbs, right-sided weakness, gait imbalance, and cerebellar symptoms. Magnetic resonance imaging demonstrated a vividly enhancing mural nodule arising from the dorsal cervicomedullary junction associated with an elongated intramedullary cystic cavity extending caudally to the C3 vertebral level, accompanied by cord edema and syringomyelia. Evaluation for von Hippel–Lindau disease was negative. The patient underwent gross total microsurgical excision through a midline suboccipital craniectomy with C1 laminectomy. Histopathological examination confirmed hemangioblastoma (WHO grade 1). The postoperative course was uneventful, with significant neurological improvement during follow-up.

**Conclusion** Primary cervicomedullary hemangioblastoma with extensive cervical cystic extension is an exceptionally rare lesion. Careful preoperative imaging evaluation, meticulous microsurgical technique, and complete tumor excision can achieve favorable neurological outcomes. Comparison with previously reported cases highlights the rarity of this presentation and supports gross total resection as the treatment of choice whenever safely feasible.

## Introduction

Hemangioblastomas grow in the blood vessels of the brain, spinal cord, and retina and are benign World Health Organization (WHO) grade 1 vascular neoplasms of the central nervous system that may produce neurological deficits through compression of adjacent neural structures.

Hemangioblastomas account for approximately 1–2% of all intracranial tumors and 7–12% of posterior fossa neoplasms. 1 They most commonly arise in the cerebellum, followed by the spinal cord. 1 Brainstem involvement is rare, representing only 3–5% of cases. 2 3 4 Tumors originating at the cervicomedullary junction with extension into the cervical spinal cord are exceedingly uncommon and are typically described only in isolated case reports. 5

Mainly intracranial hemangioblastoma occurs as a result of von Hippel–Lindau (VHL). It is an inherited genetic condition. 6 Around 20–25% of hemangioblastoma cases are caused by VHL. 7 8 It affects 1 in 36,000 people. 8 However, isolated hemangioblastoma without VHL has been reported, though rare. 1 The aim of this article is to review the clinical presentation, diagnosis, and surgical treatment of a brainstem hemangioblastoma with intradural extramedullary cervical extension.

Brainstem hemangioblastoma itself is a very rare entity, and many surgeons will not even find a single case in their lifetime of practice, and its extension into cervical region makes it even more interesting. Hemangioblastomas are classified as grade 1 benign tumors by the WHO and are known for their high vascularity. 9 Such vascular nature poses a big problem during surgical treatments due to serious difficulties in intraoperative bleeding control and differentiation of tumor vessels from normal neural structures. 2 3 The tumors predominantly arise from beneath the pia mater and are often visible only upon dural opening during surgery. 3 Thus, preoperative accurate localization is vital for complete resection and good surgical outcome. We present such a rare case and discuss its management.

## Case Report

A 39-year-old man presented in our outpatient department with a 2-year history of progressive paresthesia in both upper limbs and significant weakness in the grip of right hand, quantified as “four-fifth” strength. It was associated with episodes of headache and also complained of reduced appetite, nausea and vomiting of ingested matter, failure to maintain balance, and right-side body weakness of 1-year duration. Neurological examination revealed:

- Glasgow Coma Scale: 15/15.
- Cranial nerves: bilateral, all intact.
- Power was 4/5 on right side both in upper and lower extremities. Right grip was approximately 70% and left being normal.
- Hoffman’s sign positive.
- Position and vibration sensation were reduced on right side, and cerebellar signs were positive.

**Fig. 1:**
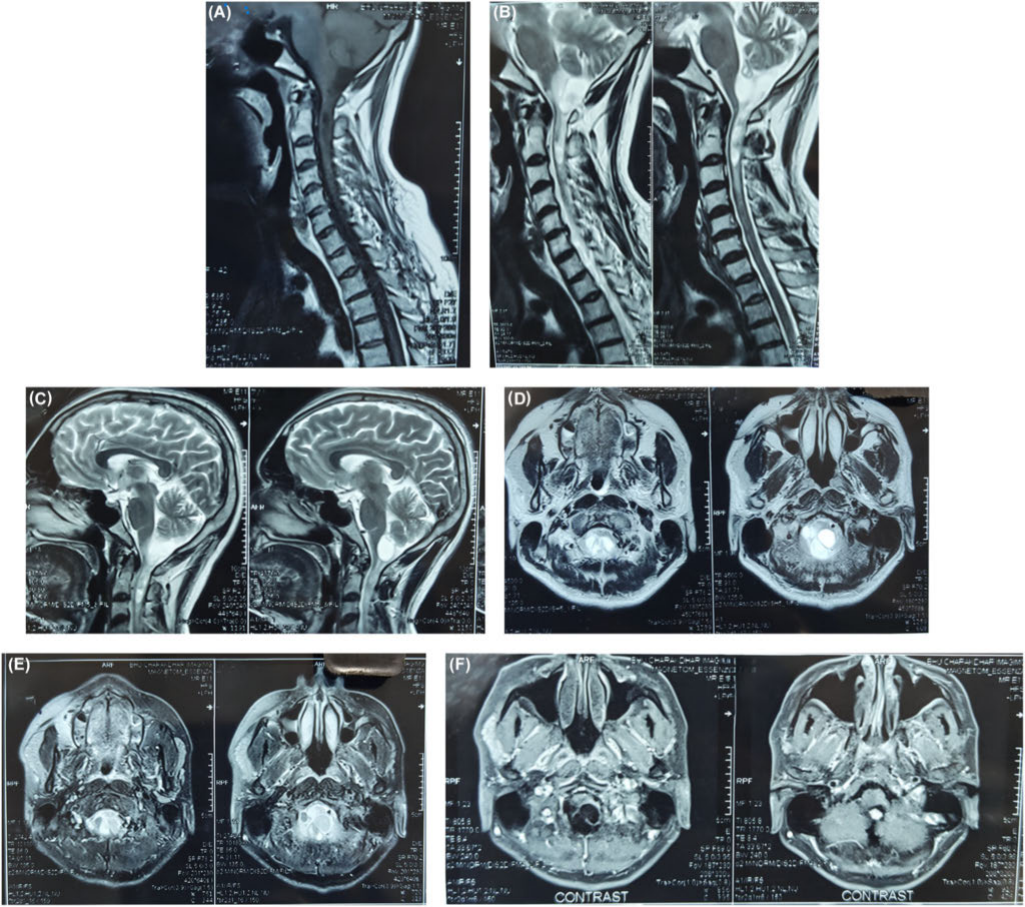
Radiodiagnosis. ( **A** ) Sagittal T1-weighted magnetic resonance image demonstrating expansion of the cervicomedullary junction and upper cervical spinal cord by a lesion extending caudally to approximately C3 vertebral level. ( **B** ) Midline sagittal and parasagittal T2-weighted image demonstrating a large cystic lesion centered at the cervicomedullary junction extending inferiorly into the upper cervical spinal cord with associated cord expansion. Also, demonstrating the dorsal relationship of the lesion to the cervicomedullary junction. ( **C** ) Sagittal T2-weighted magnetic resonance image demonstrating a well-defined hyperintense cystic component extending from the cervicomedullary junction into the upper cervical spinal cord with associated cord expansion. ( **D** ) Axial T2-weighted magnetic resonance image at the level of the cervicomedullary junction demonstrating a cystic lesion with an eccentric mural nodule causing expansion of the cervicomedullary region. ( **E** ) Axial T2 STIR (fat-suppressed) magnetic resonance image demonstrating the hyperintense cystic component and associated surrounding cord edema, clearly delineating the craniocaudal extent of the lesion. ( **F** ) Axial T1-weighted postcontrast magnetic resonance image demonstrating intense homogeneous enhancement of the mural nodule, while the associated cystic component remains nonenhancing. STIR, short tau inversion recovery.

MRI of the brain and cervical spine demonstrated a vividly enhancing mural nodule arising from the dorsal cervicomedullary junction with an associated elongated cystic cavity extending caudally to the C3 vertebral level. The solid component appeared iso- to hypointense on T1-weighted images and hyperintense on T2-weighted images, showing intense homogeneous enhancement following gadolinium administration. Multiple flow voids were present around the lesion, suggestive of a highly vascular tumor. Associated cord edema and syringomyelia were noted inferiorly ( Fig. 1A–F ).

The imaging appearance suggested a vascular lesion such as hemangioblastoma, but the differential diagnosis also included brainstem glioma due to its ill-defined nature or a metastatic tumor. However, the presence of prominent flow voids and intense homogeneous enhancement favored the diagnosis of hemangioblastoma. 2 3

Systemic evaluation for VHL syndrome was performed and included a detailed clinical and family history, ophthalmological examination, and abdominal ultrasound; all of which were unremarkable. No other central nervous system or visceral lesions were identified, effectively excluding VHL disease in this patient. 6

## Surgery

**Fig. 2:**
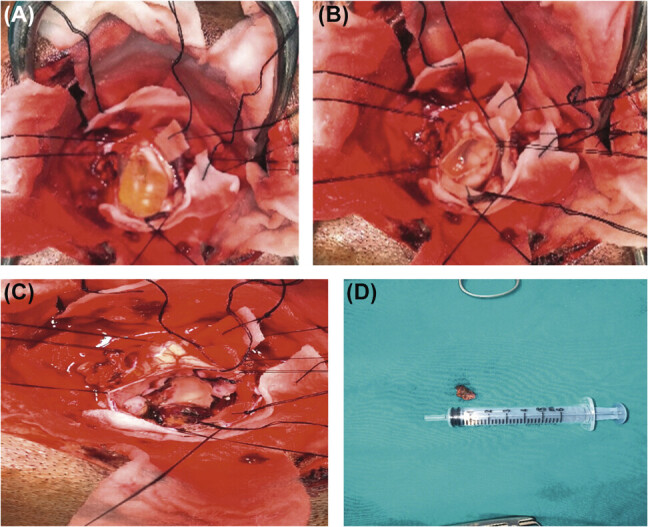
Intraoperative images. ( **A** ) Operative view after dural opening demonstrating the tense cystic component bulging through the dorsal cervicomedullary region. ( **B** ) Aspiration of the cystic component resulting in decompression of the operative field and identification of the highly vascular mural nodule following cyst decompression. ( **C** ) Circumferential microsurgical dissection of the lesion after progressive devascularization while preserving adjacent neural tissue. ( **D** ) Resected mural nodule adherent to brainstem.

The patient was prepared for elective surgery after proper workup. He was positioned prone on horseshoe, and midline suboccipital craniectomy and C1 laminectomy were done. Dura was opened, and between the cerebellar hemispheres, there was a posterior bulge on the medulla oblongata with yellowish-colored cystic component of the space-occupying lesion ( Fig. 2A ), approximately 12 mL of cystic component aspirated and the solid component reddish brown in color was densely adhered to the underlying medulla ( Fig. 2B,C ). The tumor was found to be highly vascular with ill-defined plane from the surrounding neural tissue. Circumferential devascularization was achieved prior to internal debulking, allowing for gross total excision, consistent with recommended microsurgical principles for hemangioblastoma management. 2 3 10 There was oozing blood from the tumor bed that was controlled easily with surgical and cottoned packing.

Intraoperatively, bradycardia was seen on two to three occasions during resection of the tumor that was handled well with the combined efforts of the anesthesia team.

After securing hemostasis, dura was closed in watertight fashion and reinforced with fibrin sealant. Muscle, fascia, and skin were closed layer by layer. Postoperatively, the patient was fully conscious and extubated with his vital signs being stable. The postoperative computed tomography (CT) showed complete resection of tumor with no postoperative bleeding. The patient was discharged home on 10th postoperative day after complete suture removal. On discharge, the motor and sensory function and deficit were same as preoperatively.

Follow-up visit after 1-month improvement in power of right side of body (4+/5) with grip strength (~100%) as well. Complaints of paraestheisa and headache and cerebellar symptoms also improved.

### Histopathological Examination

**Fig. 3:**
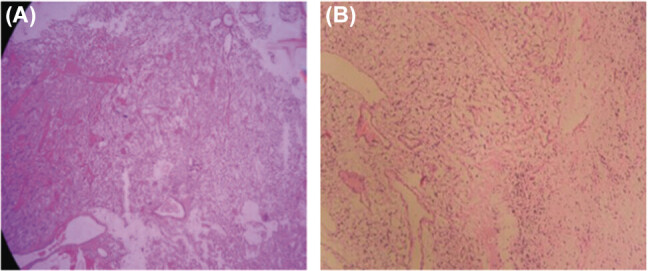
Histopathology. ( **A** ) Tumor showing congested vascular channels and interstitial stromal cells having clear cytoplasm (H&E, ×50). ( **B** ) Proliferating vascular channels with stromal cells having clear cytoplasm. Adjacent normal brain parenchyma is present (right) (H&E, ×100). H&E, hematoxylin and eosin.

- Cystic component: negative for malignant cells.
- Solid portion histopathological examination: section examined shows a tumor with surrounding brain parenchyma. The tumor is circumscribed and composed of many dilated and congested blood vessels including capillary-sized and large blood vessels admixed with many stromal cells with vacuolization ( Fig. 3A,B ). Surrounding brain parenchyma shows gliosis and many Rosenthal fibers. These findings are characteristic of hemangioblastoma and are consistent with a WHO grade 1 tumor. 9 11 Diagnosis was established based on characteristic histomorphology, and immunohistochemistry cannot be done to lack of institutional facility.

## Discussion

Hemangioblastomas of the brainstem are rare and pose significant surgical challenges due to their proximity to vital nuclei and tracts. 2 3 Extension into the cervical spinal cord further complicates management and is scarcely reported in literature. 5 These tumors are frequently associated with VHL disease, necessitating genetic evaluation and systemic screening.

### Review of Published Cases and Literature Review

Because primary brainstem hemangioblastomas with cervicomedullary or cervical extension are exceedingly uncommon, comparison with previously published cases is valuable for understanding their clinical characteristics and surgical outcomes. We therefore reviewed the available English-language literature describing surgically treated brainstem and cervicomedullary hemangioblastomas with cervical involvement. The principal demographic, radiological, operative, and outcome characteristics of these cases are summarized in Table 1 . Comparison with previously reported cases demonstrates that our patient exhibited clinical and radiological features similar to those described in the literature, while highlighting the unusual extent of caudal cystic extension and the favorable neurological outcome achieved following gross total microsurgical excision.

**Table 1:** Published cases of cervicomedullary/brainstem hemangioblastoma with cervical extension.

Author	Year	Age	Site	Cyst	Surgery	Outcome
Chuah and Tan 5	2005	42	Cervicomedullary	Yes	GTR	Good
Fukushima et al 10	2014	Multiple	Brainstem	Variable	GTR	Good
Lee et al 2	2007	Multiple	Brainstem	Yes	GTR	Improved
Present case	2026	39	Cervicomedullary	Yes	GTR	Improved

Abbreviation: GTR, gross total resection.

Review of the available literature demonstrates that most reported patients are middle-aged adults presenting with progressive myelopathy, lower cranial nerve dysfunction, or cerebellar symptoms. Magnetic resonance imaging (MRI) typically reveals a strongly enhancing mural nodule associated with a cyst or syrinx, while complete microsurgical resection remains the treatment of choice. Favorable neurological outcomes are generally achieved when gross total excision is possible. Our case is consistent with these observations but is distinguished by the extensive caudal cystic extension to the C3 level and successful complete excision without permanent neurological deterioration.

Microsurgical excision remains the gold standard treatment. 2 3 Preoperative embolization is rarely feasible due to the small caliber of feeding arteries in this region. 3 Complete resection is usually curative, as these tumors are WHO grade 1 lesions. 9

The most frequent sites of brainstem hemangioblastoma are the medulla oblongata and the fourth ventricle. Hence, hemangioblastoma of the brainstem can be classified based on these two sites into three types:

- Those tumors attached to the floor of the fourth ventricle.
- Those tumors partially embedded in the dorsal medulla oblongata.
- Type I—intramedullary. The intramedullary hemangioblastoma is the least common type. Classifying based on this site may help to assess the surgical risks and predict the clinical outcome. 3

About 70% of symptomatic brainstem hemangioblastomas are solid. 2 Cystic component of brainstem hemangioblastomas or hemangioblastomas with peritumoral cyst develops due to increased vascular permeability of the hemangioblastoma resulting in extravasation of a plasma ultrafiltrate into the tumor interstitial spaces. 3 MRI with contrast is the investigation of choice for both the diagnosis and follow-up of hemangioblastoma and is usually sufficient for preoperative evaluation. 2 CT angiography is recommended for large solid tumors, as it will allow a detailed study of the vascular anatomy and will help while planning of a surgical strategy to prevent catastrophic intraoperative bleeding. 3 10

## Conclusion

Brainstem hemangioblastoma with cervical spinal cord extension is an extremely rare entity. Despite its formidable surgical complexity, gross total resection can be safely achieved with careful technique, resulting in favorable neurological outcomes. We have reported such a case that was managed surgically with good outcome. By increasing the availability of advanced neuroimaging and with improved microsurgical techniques, complete and safe resection of rare and difficult brain lesions like brainstem hemangioblastomas is possible in resource-limited setups.

## References

[1] Mills S A Oh M C Rutkowski M J Sughrue M E Barani I J Parsa A T Sporadic hemangioblastomas of the central nervous system: clinical characteristics and outcomes J Neurosurg 2012 117 04 632 640

[2] Lee D K Choe W J Chung C K Kim H J Brainstem hemangioblastoma: surgical considerations and outcomes J Clin Neurosci 2007 14 07 670 675

[3] Roonprapunt C Lanzino G Couldwell W T Brainstem hemangioblastomas: review of surgical management Neurosurg Focus 2003 15 05 E11

[4] McCormick P C Torres R Post K D Stein B M Intramedullary ependymoma and hemangioblastoma of the spinal cord: management and outcome Neurosurgery 1990 26 05 880 887 2319309 10.3171/jns.1990.72.4.0523

[5] Chuah K L Tan K K Hemangioblastoma of the cervicomedullary junction: a case report and review of the literature Spine 2005 30 21 E640 E643

[6] Lonser R R Glenn G M Walther M von Hippel-Lindau disease Lancet 2003 361 9374 2059 2067 12814730 10.1016/S0140-6736(03)13643-4

[7] Conway J E Chou D Clatterbuck R E Brem H Long D M Rigamonti D Hemangioblastomas of the central nervous system in von Hippel-Lindau syndrome and sporadic disease Neurosurgery 2001 48 01 55 62discussion 62–63 11152361 10.1097/00006123-200101000-00009

[8] Neumann H P Wiestler O D Clustering of features of von Hippel-Lindau syndrome: evidence for a complex genetic locus Lancet 1991 337 8749 1052 1054 1673491 10.1016/0140-6736(91)91705-y

[9] Louis D N Perry A Reifenberger G The 2016 World Health Organization classification of tumors of the central nervous system: a summary Acta Neuropathol 2016 131 06 803 820 27157931 10.1007/s00401-016-1545-1

[10] Fukushima T Sakamoto S Tsugu H Surgical strategies for brainstem hemangioblastomas World Neurosurg 2014 82 06 e825 e83424056093

[11] Burger P C Scheithauer B W Tumors of the Central Nervous System Washington, DC Armed Forces Institute of Pathology 2007

